# Role of the Tomato *Non-Ripening* Mutation in Regulating Fruit Quality Elucidated Using iTRAQ Protein Profile Analysis

**DOI:** 10.1371/journal.pone.0164335

**Published:** 2016-10-12

**Authors:** Xin-Yu Yuan, Rui-Heng Wang, Xiao-Dan Zhao, Yun-Bo Luo, Da-Qi Fu

**Affiliations:** 1 The College of Food Science and Nutritional Engineering, China Agricultural University, No. 17 Tsinghua East Road, Beijing 100083, PR China; 2 Beijing Engineering and Technology Research Center of Food Additives, Beijing Technology and Business University (BTBU), 11 Fucheng Road, Beijing 100048, People’s Republic of China; Zhejiang University, CHINA

## Abstract

Natural mutants of the *Non-ripening* (*Nor)* gene repress the normal ripening of tomato fruit. The molecular mechanism of fruit ripening regulation by the *Nor* gene is unclear. To elucidate how the *Nor* gene can affect ripening and fruit quality at the protein level, we used the fruits of *Nor* mutants and wild-type *Ailsa Craig* (AC) to perform iTRAQ (isobaric tags for relative and absolute quantitation) analysis. The *Nor* mutation altered tomato fruit ripening and affected quality in various respects, including ethylene biosynthesis by down-regulating the abundance of 1-aminocyclopropane-1-carboxylic acid oxidase (ACO), pigment biosynthesis by repressing phytoene synthase 1 (PSY1), ζ-carotene isomerase (Z-ISO), chalcone synthase 1 (CHS1) and other proteins, enhancing fruit firmness by increasing the abundance of cellulose synthase protein, while reducing those of polygalacturonase 2 (PG2) and pectate lyase (PL), altering biosynthesis of nutrients such as carbohydrates, amino acids, and anthocyanins. Conversely, *Nor* mutation also enhanced the fruit’s resistance to some pathogens by up-regulating the expression of several genes associated with stress and defense. Therefore, the *Nor* gene is involved in the regulation of fruit ripening and quality. It is useful in the future as a means to improve fruit quality in tomato.

## Introduction

The tomato (*Solanum lycopersicum*) is an important model to study climacteric fruit ripening because it has advantages, such as a simple genetic background, efficient genetic transformation method, fully sequenced genome, and large agricultural acreage [1, 2]. Its fruit constitutes an important part of a healthy human diet because it contains high levels of nutrients, such as lycopene, vitamins, minerals, *etc*. Quality enhancement and nutrient synthesis in tomato fruits are closely related to its ripening process, which is regulated by various genes and environmental conditions [3, 4]. For example, lycopene is present at extremely low levels in green tomato fruit, but its synthesis proceeds rapidly and results in high levels of lycopene in mature fruit. Study of the mechanism of fruit ripening can clarify the relationship between quality enhancements and ripening in fruits; these results mean that we can change tomato fruit nutrition by controlling *Nor* gene to improve human health.

Early studies on the ripening mechanism of climacteric fruit mainly focused on the role of ethylene and included the study of ethylene synthesis and signal pathways. Consequently, the biosynthetic pathway of ethylene is well known and has been widely reviewed. The ethylene biosynthesis pathway involves two key biosynthetic enzymes: 1-aminocyclopropane-1-carboxylate (ACC) synthases (ACS), which converts *S*-adenosyl-L-methionine (SAM) to ACC, and ACC oxidase (ACO), which further converts ACC to ethylene [5–7]. By controlling the key genes of ethylene biosynthesis in climacteric fruit, the synthesis of ethylene can be effectively reduced, which significantly inhibits fruit ripening [6]. Ethylene, upon synthesis in the fruit, regulates the expression of genes associated with ripening through signal transduction pathways [8]. The screening and identification of ethylene response factors is key to understanding the mechanism by which ethylene regulates ripening, senescence, and response to stress. Several genes that regulate tomato ripening through ethylene signal transduction have been identified, including *ethylene insensitive 2* (*SlEIN2)*, *ethylene insensitive 3 (SlEIN3)*, *ethylene response factor 6 (SlERF6)* and *constitutive triple response 1* (*SlCTR1)* [9, 10].

Over the long course of the development and evolution of the tomato plant, there have been some genes whose natural mutants have a significant effect on fruit ripening, such as *Ripening-inhibitor* (*Rin*) [11], *Colorless non-ripening* (*Cnr*) [12], and *Never*-*ripe* (*Nr*) [13]. The mutants *Green-ripe (Gr)* [14] and *Non-ripening* (*Nor*) [2] exhibit an abnormal ripening phenotype including higher firmness, reduced lycopene accumulation, *etc*. However, cloning and identification of responsible genes were challenging before the entire genome of the tomato plant was sequenced. Several decades were required to clone and identify the function of these genes in tomato fruit. *Nr* encodes one ethylene receptor, and mutations in this gene lead to inhibition of the ethylene signal pathway and therefore to inhibition of normal ripening induced by ethylene [13]. *Cnr* is a squamosa promoter binding protein (SBP) that results in colorless fruit [12]. The *Gr* mutant results from a 334-bp deletion in a gene that has an unknown function [14]. The *Nor* mutation was identified as a mutation in the gene encoding a member of *NAC* transcription factor family, which plays important roles in diverse physiological processes during development, including the stress response, flowering, and senescence [15]. Currently, the regulation mechanisms of *Nr*, *Cnr* and *Rin* have been made relatively clear, whereas regulation of *Nor* is less well known. Due to the lack of a tomato mutant library, such as that available for *Arabidopsis thaliana*, the identification and characterization of ripening-associated mutants is still an effective method that is used to understand fruit ripening.

Proteomics is a powerful tool to study protein profiles in a sample. This tool has been previously used to screen the ripening-associated protein profile to study fruit ripening at the protein level [16, 17]. Using this method, the synthesis of aroma-generating compounds and the ubiquitin process of fruit ripening were found to be regulated by the *Rin* gene [16, 17].

Here, we used the iTRAQ technique to screen differentially abundant proteins in the *Nor* mutant compared with wild-type tomato fruit. The proteomic data were integrated into a network containing several metabolic or regulatory pathways related to ethylene synthesis, softening, pigment accumulation, and nutrition synthesis in *Nor* mutant fruit to elucidate the involvement of the *Nor* gene in regulating physiological and metabolic pathways, especially those related to the enhancement of fruit quality in tomato.

## Materials and Methods

### Plant materials

The seeds of wild type (WT) tomato (*Solanum lycopersicum* ‘*Ailsa Craig*’) and its late-ripening mutant *Nor* were germinated and grown in the same green house at 25°C with 75% relative humidity under a16 h light/8 h dark regime. Flowers were gently shaken with an electronic toothbrush at full-boom to obtain pollen and were tagged one day post anthesis (DPA). Fruit samples were harvested at approximately 44 DPA when the WT fruit were at the breaker stage (BK). Immediately after harvesting, the pericarps were collected, snap-frozen in liquid nitrogen, and stored at—80°C until use.

### Plasmid construction and virus-induced gene silencing (VIGS) assay

The tobacco rattle virus (TRV)-based vectors pTRV1 and pTRV2 were used for a VIGS of the tomato genes. A 540-bp fragment of the *Nor* gene corresponding to bases 617–1156 of the *Nor* gene sequence (NM_001247723.2) and a 421-bp fragment of the *phytoene desaturase* (*PDS)* gene corresponding to bases 1324–1744 of the *PDS* gene sequence (XM_010320112.1) were PCR-amplified from tomato cDNA using the primers listed in S1 Table. The resulting products were cloned into pTRV2 to form pTRV2-*Nor* or pTRV2-*PDS*.

The VIGS assay was carried out as previously described [9] with minor modifications. The *Agrobacterium tumefaciens* strain GV3101 containing the TRV-VIGS vectors was grown at 28°C in Luria-Bertani medium containing 10 mM MES and 20 mM acetosyringone with appropriate antibiotics. After incubation with shaking for 24 h, the cells were harvested and resuspended in infiltration buffer (10 mM MgCl_2_, 10 mM MES, pH 5.6, 200 mM acetosyringone) to a final OD_600_ of 6.0 (for both pTRV1 or pTRV2 and its derivatives) and left to stand for 3–4 h at room temperature (20–25°C) before infiltration. Resuspensions of pTRV1 and pTRV2 or their derived vectors were mixed at a ratio of 1:1 and infiltrated into the carpopodium of the tomato fruits that were still attached to the plant at approximately 7–10 DPA with a 1-ml syringe. Tomato fruits infiltrated with pTRV2 or pTRV2-*PDS* alone were used as control.

### RNA extraction and quantitative RT-PCR analysis

Total RNA extraction from the pericarp of the fruit was conducted using an RNeasy MiniKit (QIAGEN, Gemany) excluding polysaccharides and polyphenols from the sample according to the manufacturer's instructions. The extracted RNA was reverse-transcribed into cDNA using the one-step reverse-transcription system (TransGen Biotech, China) as described in the manufacturer’s protocol.

Quantitative real-time PCR was performed using the SYBR Green PCR TOP Mix (TransGen Biotech, China) along with a BIO-RAD real-time PCR system. The reaction program was as follows: 95°C for 10 min, 40 cycles at 95°C for 15 s and 60°C for 30 s. The fluorescence signal was measured automatically during each cycle. The relative quantification of specific mRNA levels were measured using the 2^(-ΔΔCt)^ analysis method, and expression values were normalized using the *Actin* gene and then normalized against the AC. The primers used in this study are listed in S1 Table. Three independent biological replicates were analyzed for each sample.

### Protein Extraction

Five grams of fruit tissue from each sample was ground in liquid nitrogen and homogenized in Buffer A (50 mM Tris-HCl pH 8.0, 2 mM EDTA, 100 mM KCl, and 700 mM sucrose). Then, an equal volume of Buffer B (Tris-HCl pH 7.5 saturated phenols) was added, followed by homogenization for 3 min on ice and centrifugation at 15, 000 rpm for 10 min. The upper aqueous phase was removed and re-extracted using Buffer A. Proteins in the final phenol phase were precipitated overnight with four volumes of ice-cold Buffer C (saturated ammonium acetate in methanol) at—20°C. The proteins were pelleted by centrifugation and washed with ice-cold Buffer C three times and then twice in ice-cold acetone. Protein pellets were immediately solubilized in lysis buffer [8 M urea, 30 mM HEPES, 1 mM PMSF, 2 mM EDTA and 10 mM dithiothreitol (DTT)] using 5 min of sonication (pulse-on 2 s, pulse-off 3 s, power 180 W), followed by centrifugation at 20, 000 rpm for 30 min. The supernatant was collected, and DTT was added to a final concentration of 10 mM. Then, the mixture was incubated at 56°C for 1 h and immediately reduced by adding iodoacetamide (IAM) to a final concentration of 55 mM, this was followed by a 1 h incubation at room temperature in the dark. Proteins were precipitated overnight using four volumes of ice-cold acetone at—20°C and centrifuged at 20, 000 rpm for 30 min at 4°C. The precipitate was collected and diluted in 400 μl of dilution buffer to a final concentration of 50% triethyl ammonium bicarbonate (TEAB) and 0.1% SDS. The mixture was sonicated for 3 min as previously described and centrifuged at 20, 000 rpm for 30 min at 4°C. Then, the supernatant was collected and the protein concentration was determined using the Bradford assay.

### Protein digestion and iTRAQ labeling

One hundred micrograms of protein from each sample were brought with 50% TEAB and 0.1% SDS to an equal volume and digested with 3.3 μg of trypsin at 37°C overnight. Then, 1 μg of trypsin was added and the proteins were digested again at 37°C for 12 h. The digested sample was lyophilized and resuspended in 30 μl of TEAB (water: TEAB = 1:1).

The tryptic peptides were labeled using the iTRAQ-8plex reagents according to the manufacturer’s instructions. Samples of fruit tissue from the *Nor* mutant were labeled with iTRAQ tags 113, 114 and 115, and samples from the WT were labeled with iTRAQ tags 116, 117 and 118. Three independent biological replicates were performed. The incubation was processed at room temperature for 2 h, and the iTRAQ-labeled samples were subsequently vacuum dried.

### Peptide fractionation with strong cation exchange (SCX) chromatography

For SCX chromatography, iTRAQ-labeled peptides were reconstituted with Buffer A (25% ACN, 10 mM KH_2_PO_4_, pH 3.0) to a final ten-fold volume and were centrifuged at 15, 000 rpm for 10 min. The supernatant was collected and loaded onto a Phenomenex Luna SCX column (100 A). The peptides were eluted at a flow rate of 1 ml/min using Buffer B (25% ACN, 2 M KCl, 10 mM KH_2_PO_4_, pH 3.0) as follows: a gradient of 0%–5% for 1 min, 5%–30% for 20 min, and 30%–50% for 5 min; maintained at 50% for 5 min; and then maintained at 50%–100% for 5 min. The system was maintained in 100% Buffer B for 10 min before equilibrating with Buffer A for approximately 10 min until 20 min before the next injection. The elution was monitored by measuring the absorbance at 214 nm, and fractions were collected every 1 min. The eluted peptides were finally combined into 12 pooled samples and desalted on a Strata XC18 column. The fractions were dried using low-temperature centrifugation and resuspended in 1% (v/v) formic acid.

### LC-MS/MS analysis by Q-Exactive

Nano LC-MS/MS was used with a Q-Exactive MS that was interfaced with a Thermo fisher Proteome Discoverer system. Mobile phase A was water and 1% formic acid, whereas mobile phase B was acetonitrile and 1% formic acid. The mixture of peptides was loaded onto a C18 column and separated at a flow rate of 400 ml/min using a mobile phase B gradient of 5% for 10 min, 5%–30% for 30 min, 30%–60% for 5 min, and 60%–80% for 3 min; maintained at 80% for 7 min; returned to 5% for 3 min; and maintained at 5% for 7 min. The eluted peptides were detected using Q-Exactive, and MS data were acquired using a data-dependent top20 method by choosing the abundant precursor ions from the survey scan (350–20, 000 Da) using higher energy collision dissociation (HCD). Determination of the target value was based on automatic gain control (AGC). Survey scans were acquired at a resolution of 70, 000 and the resolution for the HCD spectra was set to 17, 500.

### Protein identification and quantification

Mascot version 2.3.0 and Thermo fisher Proteome Discoverer software version 1.3 were used to identify and quantify proteins. To enhance the precision of identification and quantification, only unique peptides used for protein quantification were selected. The raw MS data were first filtered using the Proteome Discoverer software with the following parameters: molecule weight of precursor ions set between 350–6, 000 Da; minimum peaks in MS/MS set to 10; and signal-to-noise threshold set to 1.5. Searches using Mascot were made against the database tomato uni4081. The following parameters were used in the database searches: tolerance for peptides and MS/MS: 15 ppm and 20 mmu, respectively; fixed modification: carbamidomethyl (C); variable modification: oxidation (M), Gln→Pyro-Glu (N-term Q), iTRAQ 8 plex(K), iTRAQ 8 plex (Y), iTRAQ 8 plex (N-term); maximum missed cleavages 1; and enzyme: Trypsin. The FDR was less than 1%. The following parameters were used for the quantification analysis using Proteome Discoverer: protein ratio type: median; minimum peptides: 1; normalization method: median; P-value<0.05; and fold change>1.4.

### Proteomic data analysis

Proteins with a 1.4-fold change and a P-value<0.05 were identified as differentially abundant proteins. Gene Ontology (GO) analysis and a Kyoto Encyclopedia of Genes and Genomes (KEGG) pathway analysis of the differentially abundant proteins were conducted using the Gene Ontology database (http://www.geneontology.org/) and the KEGG pathway database (http://www.kegg.jp/kegg/pathway.html), respectively. Based on the acquired GO terms, the WEGO (http://wego.genomics.org.cn/) was used for GO enrichment analysis and the proteins were filtered corresponding to cellular components, molecular functions and biological processes.

## Results and Discussion

### Strong inhibition of fruit ripening as a result of silencing the *Nor* gene

**Fig 1:**
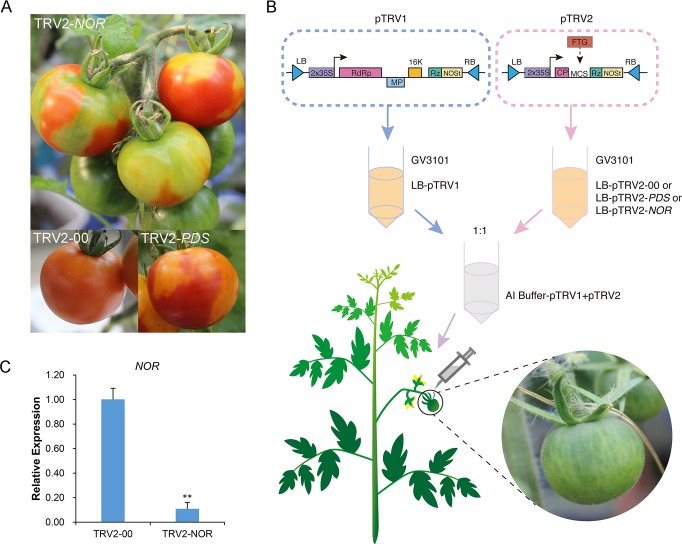
VIGS assay applied to tomato fruit. (A) Phenotype of *Nor*-silenced fruit. Vector only (pTRV2-00) and *PDS*-silenced (pTRV2-*PDS*) fruits were used as the control. (B) Flow chart of the VIGS assay for tomato fruit. To construct the pTRV2 vector, a fragment of the target gene (FTG) was inserted into the multiple cloning site (MCS). pTRV1 and pTRV2 plasmids were independently transferred to *A*. *tumefaciens* (GV3101). The cells in the LB cultures containing pTRV1 and pTRV2 were harvested and mixed at a ratio of 1:1 after resuspension in *Agrobacterium* infiltration buffer (AI buffer); the mixture was used to infiltrate the carpopodium of the tomato fruit attached to the plant at 7–10 DPA. (C) The silencing efficiency of the *Nor* gene in AC tomato fruit at the red-ripe stage (RR). CaMV 35S promoter (35S), nopaline synthase terminator (NOSt), coat protein (CP). Luria-Bertani medium (LB). Asterisks indicate a significant difference as determined using Student’s t-test (**, *P*<0.01).

With the completion of tomato genome sequencing, researchers soon discovered that the *Nor* mutant resulted from a mutation in a *NAC* family gene; this gene was cloned and its sequence was published in GenBank. To verify whether the fruit phenotype of the plants subjected to *Nor* gene silencing is consistent with that of the *Nor* mutant, VIGS was used [9] to suppress the *Nor* gene in the tomato plant. The 540-bp cDNA fragment of the *Nor* gene conserved region was PCR-amplified and cloned into the pTRV2 vector to generate pTRV2-*Nor* constructs. A mixture of *A*. *tumefaciens* (GV3101) cultures containing pTRV1 and pTRV2-Nor constructs at a 1:1 ratio were injected using a needle into the carpopodium of wild-type *Ailsa Craig* tomato fruits attached to the plant 7–10 days after pollination. Plants infiltrated with *A*. *tumefaciens* cultures, including pTRV1 and pTRV2-00, were used as controls (Fig 1B). The phenotype of the fruit was observed 25–35 days later. Twenty fruits (approximately 70%) injected with pTRV1 and pTRV2-*Nor* produced regions that failed to ripen normally and displayed a distinct green color, which is indicative of delayed ripening, or produced yellow (partially ripened) fruits at a time when parts of the fruits not subjected to silencing turned red. The green region, indicating inhibited ripening on the *Nor* fruit subjected to VIGS, eventually turned orange and showed signs of slow ripening. All control fruits injected with the pTRV1 and pTRV2-00 vector ripened normally (Fig 1A).

To confirm *Nor* gene suppression at the molecular level, primers specific to the *Nor* genes outside the region targeted for silencing were designed for real-time PCR. The reduction in the *Nor* transcripts in the silenced green part was 86% compared with the control fruit (in the parts that had the normal red color). Levels of the *ACT1* transcript were similar in tissues infected with pTRV2-*Nor* and pTRV2-00 vector constructs (Fig 1C). Tomato fruit infected with pTRV1 and pTRV2-*PDS* turned yellow, as observed for the positive control. Tomato fruits infected with pTRV1 and pTRV2-00 turned red as observed for the empty control (Fig 1A). These results indicate that the *Nor* gene plays an important role in controlling the ripening of tomato fruits, but its mechanism of regulating fruit ripening was not clear. This was further studied in the next set of experiments.

### Primary data analysis and protein identification in the *Nor* mutant

**Fig 2:**
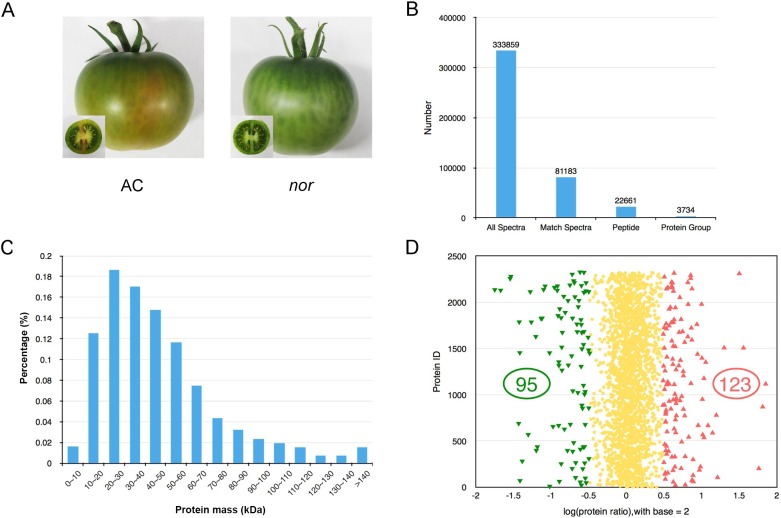
Overview of the identified proteins in the *Nor* mutant fruit. (A) Phenotype of *AC* and *Nor* tomato fruit at 44 DPA for the iTRAQ study. (B) The number of all spectra, match spectra, peptides, and protein groups from the iTRAQ analysis. (C) Identified proteins were grouped according to their protein mass. (D) Abundance distribution of the identified proteins in *Nor* mutant versus AC fruit across three biological replicates. Green triangles indicate significantly decreased proteins (n = 95), red triangles represent accumulated proteins (n = 123), and yellow spots represent the non-significant proteins.

To further unravel the mechanism of *Nor* regulation of fruit ripening at the level of protein, the iTRAQ technique will be performed using WT and *Nor* mutant tomato fruit. First, we need to make sure that *Nor* expression cannot be detected in *Nor* mutant. Total RNA from the break stage (BK) of the WT and *Nor* mutant tomato fruit was prepared and used to reverse transcript to cDNA for *Nor* gene RT-PCR. The results showed that the expression of the *Nor* gene was barely detected in the *Nor* mutant (S1 Fig). This indicates that the material can be used for the iTRAQ analysis of the *Nor* mutant. Next, total proteins were extracted from the pericarp tissue of *Nor* mutant and WT fruits and were analyzed using the iTRAQ technique at BK (Fig 2A).

After pooling data from three biological replicates, a total of 333, 859 spectra were generated from the iTRAQ analysis using the control and *Nor* mutant fruits at BK. Mascot identified a total of 81, 183 spectra matched to known spectra, which were further matched to 22, 661 peptides and finally matched to 3, 734 proteins with a FDR of less than 1% (Fig 2B). The distribution of mass coverage of proteins is provided in Fig 2C and 2D.

When screening for differentially abundant proteins between WT and the *Nor* mutant, a protein species was considered differentially accumulated if it exhibited a fold change>1.40 and a P-value<0.05. Using these two criteria, 218 differential abundant protein species including 131 uncharacterized proteins were screened from the *Nor* mutant sample compared with the wild type, of which 123 proteins were up regulated and 95 were down regulated (Fig 2D). Detailed information is provided in Table 1 and in S2 Table.

**Table 1:** Differentially expressed proteins identified in the *Nor* mutant fruit using iTRAQ LC-MS/MS.

Accession	Description	Accession Number	Ratio	P-value^a^
***Ethylene biosynthesis***				
Solyc02g036350.2	1-aminocyclopropane-1-carboxylate oxidase	A4ZYQ6	0.66	3.57E-24
Solyc07g049530.2	1-aminocyclopropane-1-carboxylate oxidase 1	P05116	0.53	5.71E-48
Solyc09g089580.2	1-aminocyclopropane-1-carboxylate oxidase homolog	P10967	0.30	5.85E-232
Solyc03g095900.2	E8 protein homolog	K4BJ12	0.51	2.31E-17
Solyc01g101060.2	S-adenosylmethionine synthase 1	P43280	1.53	8.07E-45
Solyc09g008280.1	S-adenosylmethionine synthase 3	P43282	1.47	1.59E-31
***Cell Wall Metabolism***				
Solyc01g087210.2	Cellulose synthase	K4AYB2	1.96	2.72E-04
Solyc12g056580.1	Cellulose synthase	K4DFY9	1.64	7.03E-08
Solyc03g111690.2	Pectate lyase	K4BK32	0.37	3.05E-08
Solyc10g080210.1	Polygalacturonase-2	P05117	0.31	9.58E-78
***Flavonoid biosynthetic process***				
Solyc09g091510.2	Chalcone synthase 1	K4CWH7	0.55	1.15E-08
Solyc05g052240.2	Chalcone-flavonone isomerase family protein	K4C1Q5	0.68	5.85E-09
Solyc05g047530.2	Trans-cinnamate 4-monooxygenase (Fragment)	Q42895	0.62	1.09E-04
Solyc10g085230.1	Glycosyltransferase	K4D3V7	0.46	6.59E-24
Solyc07g043120.1	Glycosyltransferase	K4CEH2	0.66	3.05E-04
Solyc09g092500.1	Glycosyltransferase	K4CWS6	0.68	4.66E-13
***Carotenoid biosynthetic process***				
Solyc12g098710.1	15-cis-ζ-carotene isomerase (Fragment)	A0A097PQ02	0.49	6.35E-07
Solyc03g031860.2	Phytoene synthase	Q2LDA3	0.59	7.77E-04
***Carbohydrate metabolic process***				
Solyc01g008710.2	Mannan endo-1,4-β-mannosidase 4	Q8L5J1	0.35	3.86E-11
Solyc11g008720.1	β-glucosidase	K4D5D2	0.53	8.06E-10
Solyc12g008840.1	β-galactosidase	E3UVW6	0.54	1.21E-02
Solyc03g083910.2	Acid invertase	D5L5R0	0.65	3.48E-59
Solyc07g045540.2	Glucose-6-phosphate 1-dehydrogenase	K4CEW0	1.41	1.55E-02
Solyc04g081300.2	Endoglucanase	K4BVK9	1.49	3.62E-06
Solyc08g083320.2	Starch synthase, chloroplastic/amyloplastic	K4CPX6	1.54	2.63E-36
Solyc04g082630.2	Glyceraldehyde-3-phosphate dehydrogenase	K4BVZ0	1.57	2.11E-23
Solyc05g005080.2	Endoglucanase	K4BW39	2.02	1.11E-04
Solyc06g050130.2	Alpha-galactosidase	K4C5G0	3.61	2.72E-02
Solyc10g017600.2	Hexosyltransferase	K4CYN8	1.42	1.04E-04
Solyc04g009030.2	Glyceraldehyde-3-phosphate dehydrogenase	K4BP59	1.44	2.87E-26
Solyc01g007330.2	Ribulose bisphosphate carboxylase large chain	P27065	1.83	9.50E-136
***Amino acid metabolic process***				
Solyc12g088220.1	Branched-chain-amino-acid aminotransferase	K4DGP3	0.56	2.16E-03
Solyc08g014130.2	Isopropylmalate synthase	K4CJ46	0.6	1.33E-11
Solyc07g008530.1	Tyrosine—tRNA ligase	K4CBX2	1.58	2.37E-03
Solyc12g099930.1	Hop-interacting protein THI032	G8Z261	1.84	2.47E-21
Solyc12g006470.1	Gamma aminobutyrate transaminase 2	K4DBI4	2.00	3.25E-10
Solyc01g005560.2	Isocitrate dehydrogenase [NADP]	K4ASC2	0.69	2.23E-33
Solyc09g092380.2	Adenosylhomocysteinase	K4CWR4	0.64	3.28E-98
Solyc01g005560.2	Serine hydroxymethyltransferase	K4BCV4	1.78	2.93E-82
***Lipid metabolic process***				
Solyc01g099190.2	Linoleate 9S-lipoxygenase B	P38416	0.52	0.00E+00
Solyc01g099160.2	Lipoxygenase	Q9FT17	0.61	5.92E-50
Solyc01g006540.2	Lipoxygenase	K4ASM0	0.63	2.84E-10
Solyc01g099180.2	Lipoxygenase	K4B0V5	0.63	1.99E-04
Solyc02g085870.2	3-ketoacyl-CoA synthase	C6KH60	0.69	1.42E-02
Solyc03g005020.2	Lipase	K4BDS6	0.41	1.06E-03
***Defense response***				
Solyc01g060020.2	Glucan endo-1,3-β-glucosidase B	Q01413	0.47	3.14E-63
Solyc01g111080.2	Snakin-2	E5KBY0	1.86	1.28E-03
Solyc08g080640.1	Protein NP24	P12670	0.47	6.19E-04
Solyc00g174340.1	Pathogenesis-related protein	Q0H8U4	0.66	7.79E-22
Solyc10g055810.1	Basic 30 kDa endochitinase	Q05538	0.53	1.82E-16
Solyc04g071900.2	Peroxidase	K4BTH7	0.66	1.89E-13
Solyc01g090350.2	Non-specific lipid-transfer protein	K4AYX6	3.39	5.13E-11
Solyc01g090360.2	Non-specific lipid-transfer protein	K4AYX7	1.66	1.26E-15
***Response to stress***				
Solyc05g014280.2	Small heat shock protein	Q8L470	1.42	1.57E-48
Solyc08g078700.2	Mitochondrial small heat shock protein	O80432	1.54	1.75E-68
Solyc06g076520.1	17.7 kD class I small heat shock protein	Q9SYU8	1.55	1.30E-12
Solyc08g062450.1	Class II small heat shock protein Le-HSP17.6	Q96489	1.81	9.50E-28
Solyc08g062340.2	17.4 kD class I small heat shock protein	K4CL22	1.93	1.36E-30
Solyc03g111720.2	Methionine sulfoxide reductase A	G3JX11	0.38	6.17E-40
***Phenylpropanoid metabolic process***				
Solyc10g086180.1	Phenylalanine ammonia-lyase	K4D451	0.55	7.41E-05
Solyc09g007900.2	Phenylalanine ammonia-lyase	K4CQH9	0.67	5.84E-05
Solyc08g005770.2	Alcohol acyl transferase	Q6QLX4	0.34	1.15E-73
***Signaling***				
Solyc01g097770.2	Phototropin-2	A7LI54	1.57	7.83E-13
Solyc11g072630.1	Mitogen-activated protein kinase	E2GLN8	1.54	4.22E-02
Solyc01g108560.2	Carboxylesterase 1	K7SGP9	0.41	9.75E-24
Solyc05g051750.2	Systemin	P27058	1.45	7.43E-13
Solyc02g077880.2	Auxin repressed/dormancy associated protein	Q0PY39	1.52	1.25E-11
***Protein degradation***				
Solyc01g087820.2	SBT4B protein	Q9ZS44	0.68	1.76E-04
Solyc08g079880.1	P69C protein	O65834	0.69	6.45E-05
***Transportation related***				
Solyc11g069700.1	Elongation factor 1-alpha	K4DAC6	0.64	6.89E-10
Solyc10g051390.1	Glycine rich RNA binding protein 1a	L7Q568	0.71	3.11E-22
Solyc01g094690.2	Plasmamembrane intrinsic protein 12	K4AZL0	1.57	1.30E-11
Solyc12g010320.1	Temperature-induced lipocalin	Q38JD4	0.62	2.99E-102
Solyc07g006730.2	Protein DETOXIFICATION	K4CBE4	1.43	1.61E-02
***Nucleic acid binding***				
Solyc02g069260.2	AGO2A2	K4B7Q8	0.67	3.67E-24
Solyc03g098280.2	Protein argonaute	K4BJP3	1.94	2.53E-02
Solyc01g079870.2	CONSTANS interacting protein 2b	Q2VY17	0.69	1.76E-02
***Pyrimidine metabolic process***				
Solyc07g065890.2	Uridine kinase	K4CHR5	1.53	4.60E-16
Solyc01g100030.2	P18 protein	K4B137	1.41	1.59E-10
***Anion binding***				
Solyc04g055170.2	Annexin	K4BSR4	3.51	6.21E-67
Solyc08g005630.2	Long-chain-alcohol oxidase	R9R6I9	0.65	9.27E-09
***Transferase Activity***				
Solyc05g011890.1	Sulfotransferase	K4BXR2	1.67	6.52E-04
Solyc03g121830.1	Glycylpeptide N-tetradecanoyltransferase	K4BMY2	1.59	2.89E-16
***Nutrient reservoir activity***				
Solyc08g080490.2	Seed storage protein Lec2SA1 small chain (Fragment)	Q7M1T1	1.81	3.18E-05
Solyc09g082340.2	Vicilin	B0JEU3	1.72	2.54E-139
***Ripening regulated***				
Solyc04g072160.2	Ripening regulated protein DDTFR8	K4BTK3	1.40	2.56E-09
***Microtubule-based process***				
Solyc04g081490.2	β-tubulin	Q38MV0	1.53	1.43E-31

^a^ *p* values were calculated using one-way ANOVA.

### Bioinformatic analysis of differentially abundant protein species (DAPS) identified using iTRAQ

**Fig 3:**
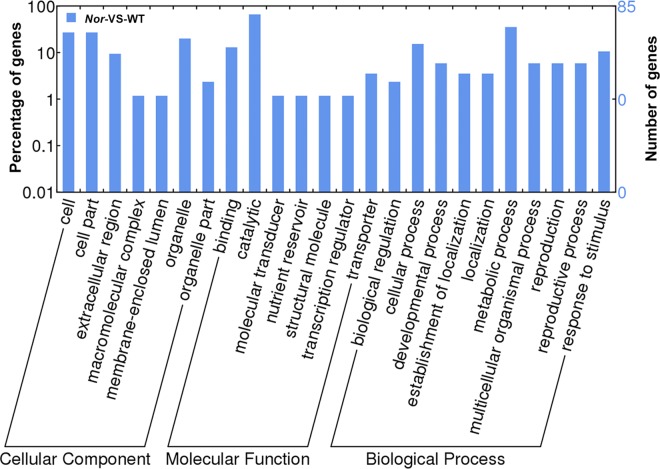
Functional classification of the differentially displayed proteins between *Nor* mutant and wild-type AC tomato fruit at 44 DPA according to WEGO.

To identify significantly enriched GO functional groups of DAPS, GO annotation was carried out. A total of 87 DAPS (40%) between the *Nor* mutant and control were classified into 24 functional groups (Fig 3), of which biological processes accounted for 10 GO terms (the most representative were “metabolic process”), cellular components accounted for 7 GO terms (the most representative were “cell and cell part”), and molecular functions accounted for 7 GO terms (the most representative was “catalytic”).

**Fig 4:**
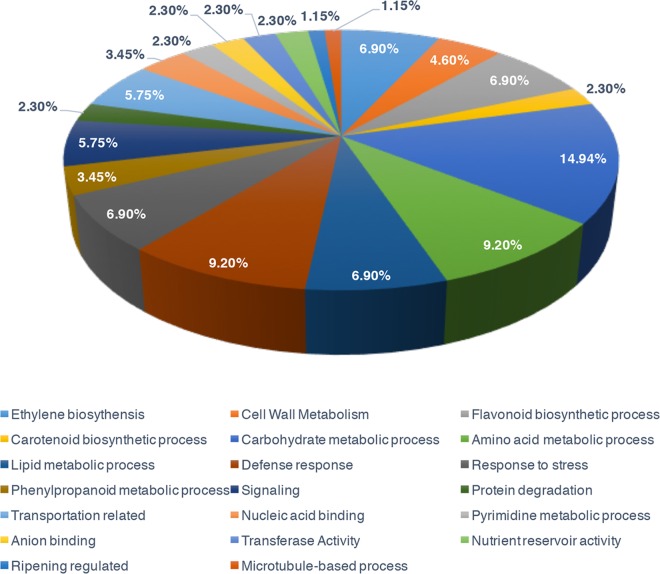
Functional categorization of the GO biological processes in the differentially expressed proteins of *Nor* mutant fruits.

To further investigate the biological functions of these proteins, 87 (40%) were mapped to 20 pathways in the KEGG database (Table 1). “carbohydrate metabolic process” was the most represented pathway followed by “defense response” and “amino acid metabolic process” (Fig 4).

**Fig 5:**
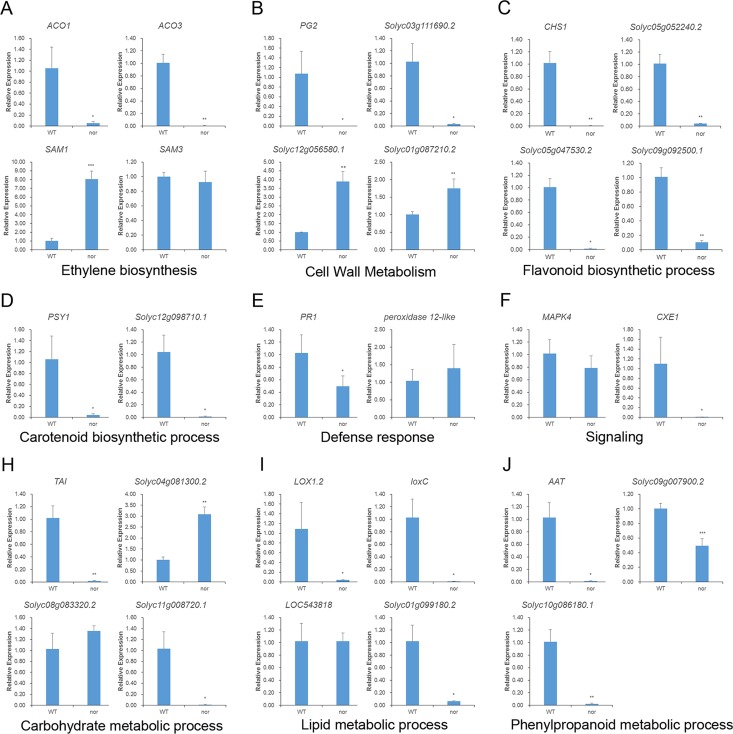
Gene expression levels in *Nor* mutant tomato fruit at 44 DPA. (A-J) Expression level of ethylene biosynthesis, cell wall metabolism, flavonoid biosynthetic process, carotenoid biosynthesis process, defense response, signaling, carbohydrate metabolic process, lipid metabolic process, and phenylpropanoid metabolic processes in *Nor* mutant tomato fruit. Gene expression levels were detected using RT-PCR from three biological replicates using three independent experiments. Asterisks indicate significant differences as determined by Student’s *t*-test (*, *P*<0.05; **, *P*<0.01; ***, *P*<0.001).

To verify whether the differentially abundant proteins have the same tendency as the expression of their target genes at the transcript level, real-time PCR was used to detect the expression of target genes in the *Nor* mutant and wild-type tomato fruit. Because we wanted to examine the pathways related to quality formation and ripening of *Nor* mutant samples (which we discuss in the next section), the target genes selected for analysis included genes related to ethylene biosynthesis (4), fruit softening (4), pigment synthesis (6), nutrient synthesis (11), and stress and defense pathways (2). iTRAQ data for the proteins encoded by these genes indicated that some were up regulated, such as the cellulose synthase gene, whereas some were down regulated, such as *PSY1* and *CHS1*. The results of real-time PCR indicate that the trend of most of these gene expressions was consistent with that of the differentially abundant proteins (Fig 5), which indicates the reliability of our iTRAQ data and that these data can be used for further analysis.

### Ethylene biosynthesis

**Fig 6:**
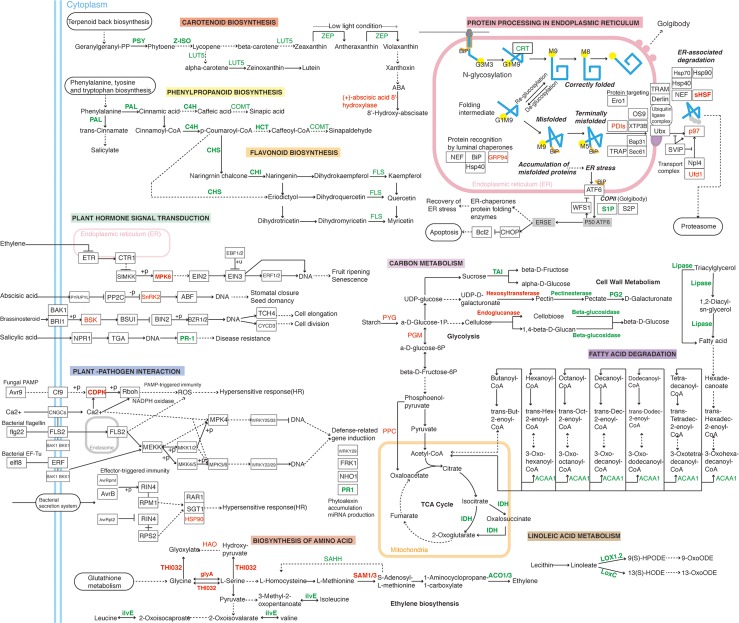
Schematic diagram of the representative differentially abundant proteins in the metabolic pathways of *Nor* mutant tomato fruit. The metabolic pathways were constructed based on the KEGG pathway analysis. Dashed arrows represent the multiple steps between two compounds, the red colored text indicates the accumulated proteins, the green colored text indicates decreased expressed proteins in the *Nor* mutant tomato fruits, and the boldfaced text represents the proteins listed in Table 1.

Ethylene is a key factor promoting the ripening of climacteric fruits, such as tomatoes [4]. Methionine is the direct precursor of the ethylene biosynthesis pathway; ACC synthase and ACC oxidase catalyze two key steps that are coded by the gene family [6]. The expression of the *SlACS2* and *SlACO4* genes increases in response to considerable ethylene synthesis during fruit ripening [6]. In addition, SAM synthase (SAMS) catalyzes ethylene synthesis from methionine in the first step of the reaction; silencing this gene can effectively reduce ethylene biosynthesis and considerably alter the ripening of tomato fruit compared with the control [6]. Our protein data indicated that the relative abundances of the four *ACO* genes in *Nor* tomato fruit were 66%, 53%, 30%, and 51%, respectively, compared with the control fruits, whereas the abundance of two *SAMS* increased by 153% and 147%, respectively. Real-time PCR revealed that the expression of the four *ACO* genes in the *Nor* mutant was significantly less than that in the control fruit. Ethylene synthesis barely occurred in the *Nor* tomato fruit (data not shown). These results indicate that the *Nor* gene regulates the expression of the *SAM1* and *ACO* genes, which are involved in the regulation of fruit ripening (Fig 6).

### Fruit softening

With the ripening of tomato fruit, the firmness of the fruit also changes significantly, primarily due to the reduction in cellulose synthesis and pectin degradation. Firmness is an important factor affecting the storage period and quality of fruit after fruit harvest [4]. The process of fruit ripening is accompanied by a series of changes in the expression of genes associated with the synthesis and degradation of cell wall substances.

Cellulose is the main structural component of the plant cell wall and is typically cross-linked with hemicellulose, pectin, and lignin, which all enhance fruit firmness [18]. Plant cellulose synthases belong to the family of glycosyltransferases, which includes proteins involved in cellulose biosynthesis [19]. The two cellulose synthase proteins in *Nor* mutant are more abundant than that of wild type: 1.96- and 1.64-fold, respectively, respectively. This indicates that the *Nor* mutant can synthesize considerably more cellulose than the wild type, which has positive implications for fruit firmness.

Polygalacturonase, which is also known as PG, is an enzyme that hydrolyzes the alpha-1, 4 glycosidic bonds between galacturonic acid residues [20]. The activity of the endogenous plant PGs results in the softening and sweetening of the fruit during the ripening process [21]. During the ripening process, *PG* gene expression significantly increases, promoting the softening of the fruit. Pectate lyase is an enzyme that is involved in the maceration and soft rotting of plant tissue and is responsible for the eliminative cleavage of pectate, yielding oligosaccharides with 4-deoxy-α-D-mann-4-enuronosyl groups at their non-reducing ends. The protein is maximally expressed in the late stages of fruit ripening [22]. The expression of *pectate lyase* (*PL*) genes has been suggested to be related to a requirement for fruit softening. Our iTRAQ data show that the levels of PG and PL protein in the *Nor* mutant were significantly less than in the control fruit: 0.31- and 0.37-fold less, respectively.

Based on these data, we suggest that the *Nor* mutation significantly enhances tomato fruit firmness by promoting the expression of the cellulose synthase gene and inhibiting the expression of the *PG* and *PL* genes (Fig 6).

### Fruit nutrition

The process of fruit ripening is accompanied by the synthesis and degradation of specific nutrients that provide good quality food for humans. The changes that occur during fruit ripening include lycopene synthesis, chlorophyll degradation, synthesis of sugars, starch degradation, flavor compound synthesis, and organic acid degradation, as well as changes in amino acids and synthesis of secondary metabolites [3]. As a result of the silencing or mutation of key ripening-associated genes, such as *Rin*, *Cnr*, *apetala 2a (AP2a)*, *tomato agamous-like1* (*TAGL1)* and *fruitfrull1 (FUL1/TDR4)*, the fruit ripening process was significantly inhibited [2]. Synthesis of the nutrients described above was also considerably affected. Silencing of the *TAGL1* gene resulted in a decrease in the lycopene, lutein and chlorophyll contents of tomato fruit [23]. Tomato fruit with antisense *TDR4* reduced the lycopene levels by down regulating the expression of *1-deoxy-D-xylulose 5-phosphate synthase 1 (DXS1)*, which affected lipid and cuticle metabolism, resulting in fruit wilting and a significant decrease in free glutamic acid by up regulating the expression of the glutamic acid decarboxylase gene [24]. *Rin* regulates the *tomato lipoxygenase* (*TomloxC)*, *polyneuridine aldehyde esterase (PNAE)*, *phosphoglycerate kinase (PGK)* and *alcohol dehydrogenase 2 (ADH2)* genes in the plant lipoxygenase (LOX) pathway, which affect flavor compound synthesis in tomato fruit [16]. The *golden-like2* (*GLK2*) gene regulates the synthesis of fruit nutrients, such as lycopene, β-carotene, lutein, ascorbic acid, fructose, glucose, sucrose, and starch, as well as the synthesis of other nutrients. Together, the data indicate that ripening-associated transcription factors regulate the synthesis of important nutrients during the tomato ripening process [25]. Mutation in the *Nor* gene hampers the ripening of the fruit. To determine whether this mutation also affects the quality of the fruit with respect to the levels of nutrients, we analyzed the levels of differentially abundant proteins in *Nor* mutant and wild-type fruit. The mutation in the *Nor* gene was found to affect the synthesis of several nutrient biomolecules and enzymes involved in various metabolic processes (Table 1, Fig 6), including carbohydrates (13), lipids (6), amino acids (8), secondary metabolites of brass (3), *etc*. The real-time PCR results of these genes encoding target proteins were consistent with the observed trend of protein expression. The results showed that the *Nor* gene was involved in the synthesis of nutrients during fruit quality enhancement and played an important role in the ripening and quality enhancement of tomato fruit.

### Pigment synthesis

The pigmentation of ripe tomato fruit is gradually generated by the accumulation of pigments, such as carotenoids and flavonoids, along with the degradation of chlorophyll [26]. In our study, the protein level of eight pigmentation related proteins was significantly less in *Nor* mutant fruits compared with the controls; two of these proteins, PSY1 and Z-ISO, take part in carotenoid biosynthesis: PSY1 is an enzyme catalyzing the synthesis of phytoene from geranylgeranyl pyrophosphate (GGPP). Down-regulation of the *PSY1* gene by transgenic technology or VIGS eliminates normal carotenoid formation [27]. *Z-ISO* gene is highly expressed in tomato fruit, and silencing this gene using VIGS leads to a dramatic reduction in lycopene content [27]. These results indicate that carotenoid biosynthesis was affected by *Nor* gene (Fig 6).

In addition, six proteins related to flavonoid biosynthesis were repressed in *Nor* mutant fruit: CHS1, chalcone-flavonone isomerase family protein (CHI), *trans*-cinnamate-4-monooxygenase, and three glycosyltransferase proteins. The repression of these proteins essentially abolished the flavonoid biosynthesis pathway (Fig 6). Moreover, the biosynthesis of phenylpropanoid, which is the precursor of flavonoid, was also suppressed, as revealed using KEGG pathway analysis. This evidence suggests that *Nor* functions is a positive regulator in these processes. In a previous study, a reduction in the level of phenolics was detected in the *Nor* fruit; these observations are consistent with our analysis [28]. CHS1 protein was significantly repressed (0.55-fold as revealed by iTRAQ and 0.005 as revealed by RT-PCR) in the *Nor* mutant. *CHS1* down regulation reportedly reduces the level of yellow-pigmented naringenin chalcone, which is generally present at high levels in the epidermal cells of ripening fruit [29]. Notably, some of the ripening-associated transcription factors are suggested to function in the activation of the flavonoid pathway, such as *TDR4* and *TAGL1* [30, 31]. Both *TDR4* and *TAGL1* RNAi tomato fruit exhibited a similar orange-yellow fruit phenotype with reduced the levels of *CHS1* expression and naringenin chalcone accumulation in the cuticle. There are a few reports indicating the relationship between flavonoid biosynthesis and fruit ripening, but the mechanism is still unclear.

### Stress and Defense Response

The stress and defense responses are plant defense mechanisms that act against invasion by bacteria and fungi (biotic stress) or as adaptations to changes in environmental conditions (abiotic stress). Pathogenesis-related proteins (PRs) are defined as a family of plant proteins that are induced and accumulate in plant tissues under pathological or related situations [32]. PRs possess antimicrobial activities in vitro through hydrolytic attacks on cell walls, contact toxicity, and their putative involvement in defense signaling; PRs have been classified into 17 families [33]. Our iTRAQ data revealed that seven PRs were differently displayed in *Nor* mutant fruits, of which five proteins were depressed to various extents: glucan endo-1, 3-β-glucosidase B (0.47-fold), an osmotin-like protein (NP24) (0.47-fold), a pathogenesis-related protein (PR1) (0.66-fold), a class Ⅰ chitinase (CHI9) (0.53-fold), and peroxidase (0.66-fold). Glucan endo-1,3-β-glucosidase B and CHI9 can hydrolyze glucan or chitin, respectively, which degrade bacterial and fungal cell wall and inhabit pathogen growth [34]. Moreover, the protein level of glucan endo-1,3-β-glucosidase is reported to be regulated by *Bradyrhizobium japonicum* inoculation in seedlings under flooding [35]. NP24, which is a thaumatin-like protein in tomato fruit, has been tested as a growth inhibitor against six different fungi [36] and is up regulated in response to salt or osmotic stress [37, 38]. Peroxidase belongs to the family of class III plant peroxidases, which partake in a wide-range of physiological processes, for instance, lignin and suberin formation, cell wall component cross linking, and phytoalexins synthesis, or participate in reaction oxygen species (ROS) and reactive nitrogen species (RNS) metabolism, which “turn on” the hypersensitive response [39]. PR1 is part of the PR-1 protein family, which is in addition to the known PR-1a, PR-1b, and PR-1c proteins found in tomato. The main PR-1 proteins induced by salicylic acid (SA) or pathogens are commonly used as markers of the state of systemic acquired resistance (SAR), but their biological function is still unknown [33, 40]. In contrast, two non-specific lipid-transfer proteins (LTPs) are considerably up regulated by 3.39-fold and 1.65-fold. These proteins belong to the PR-14 family, whose members are approximately 70 and 90 amino-acid-(aa)-long cationic proteins with eight Cys residues. According to a previous study, LTPs are suggested to be extracellular proteins and promote the formation of pores in the membranes of pathogens rather than hosts cells, which inhibits the growth of pathogens, and are involved in the plant defense system [41].

Small heat shock proteins (sHSPs) weighing between 12 and 42 kDa are the most ubiquitous subgroup of heat shock protein (HSPs). They are encoded in response to heat stress and are commonly not found in normal plant tissues, whereas the accumulation of some sHSPs has been detected during plant developmental processes, for example, pollen and embryo development, seed germination, and fruit ripening [42]. Our iTRAQ data suggest that all five sHSPs protein significantly accumulated in the *Nor* mutant. HSP17.4, which is regulated by seed development and stress, is associated with seed desiccation tolerance in *Arabidopsis* [43]. HSP17.6 can be induced by *Botrytis cinerea* in red ripe tomato fruit instead of in mature green fruit, which indicates the ripening-regulated susceptibility of tomato fruit to this fungus [44]. HSP17.7 is related to the UVA, UVB, Cu^2+^, and Zn^2+^ stress responses in sunflowers [45]. Moreover, MTSHP, which is a mitochondrial small heat shock protein in tomato, has been reported to possess molecular chaperone activity *in vitro* [46]. Additionally, *viscosity 1* (*VIS1*) contributes to pectin depolymerization and juice viscosity in tomato [42], leading to speculation that other sHSPs could play a role in fruit ripening.

Snakin-2 is a member of the snakin peptide family, which is characterized by maintaining 12 cysteines in the highly conserved C-terminus, forming six disulfide bonds [47]. Overexpression of *Snakin-2* restricts pathogen invasiveness and enhances tolerance to *Clavibacter michiganensis* in transgenic tomato plants [48]. The *Snakin-2* gene is induced by wounding and responds to pathogen invasion in potatoes [49]. Snakin-2 protein harbors antimicrobial activity against bacteria and fungi, and this activity is mediated through the non-specific perforation of the microbe’s biomembrane [47, 50]. Here, Snakin-2 shows a significantly increased level (1.86-fold) in the *Nor* mutant, indicating a changed tolerance to pathogen infection.

The methionine sulfoxide reductase (Msr) family contains ubiquitous enzymes that function to protect cells against oxidative damage [51]. The two key enzymes in the Msr family are MSRA and MSRB, which can reduce the S and R epimers of methionine sulfoxide to methionine, respectively [52]. Both enzymes have been shown to defend bacteria, yeast, and plant cells against the cytotoxic effects of ROS, which prevents undue amassing of oxidized proteins and aging-associated death [53]. In tomato, the fruit ripening gene *E4*, which is strongly induced by ethylene, encodes a MSRA protein [54]. This MSRA protein displayed significantly depressed accumulation (0.38-fold), which is likely attributed to the elimination of ethylene in the *Nor* mutant fruit.

## Conclusions

Ripening is the crucial stage of fruit development that prepares the seed for reproduction and confers fruit quality traits, such as color, aroma, taste, and texture, to ultimately provide food fit for human consumption. Therefore, some important genes controlling fruit ripening will also affect fruit quality, including nutrient synthesis. The tomato fruit has several well-known ripening-repressed natural mutations, such as *Rin*, *Cnr*, *Nr*, and *Nor*. The involvement of these three genes in the regulation of fruit ripening and formation and in the development of a good-quality fruit has been extensively studied, but there is limited knowledge regarding the involvement of the *Nor* gene. Here, we analyzed differences in the abundances of proteins in *Nor* mutant and wild-type fruit using the iTRAQ proteomic technique. The results indicate that the *Nor* mutation has a significant effect on ethylene synthesis, pigment biosynthesis, softening, and nutrient biosynthesis, as well as impacts disease resistance-associated genes. Moreover, our study data show that *Nor* plays an important role in the development of fruit quality. We should, however, further verify whether the *Nor* gene, as a transcription factor, directly regulates the transcription of the genes encoding the differentially abundant proteins in the *Nor* mutant of tomato fruit.

## Supporting Information

S1 Fig *Nor* gene expression in *Nor* mutant tomato fruit. (TIF)

S1 Table Oligonucleotide primers used in the study. (XLSX)

S2 Table Differential expressed uncharacterized proteins identified in *Nor* mutant fruit by iTRAQ LC-MS/MS. (XLSX)
