# Supplementary figures and images for: Role of the Tomato Non-Ripening Mutation in Regulating Fruit Quality Elucidated Using iTRAQ Protein Profile Analysis
Source: PLoS One. 2016 Oct 12;11(10):e0164335. doi: 10.1371/journal.pone.0164335 (PMC5061430; doi:10.1371/journal.pone.0164335)

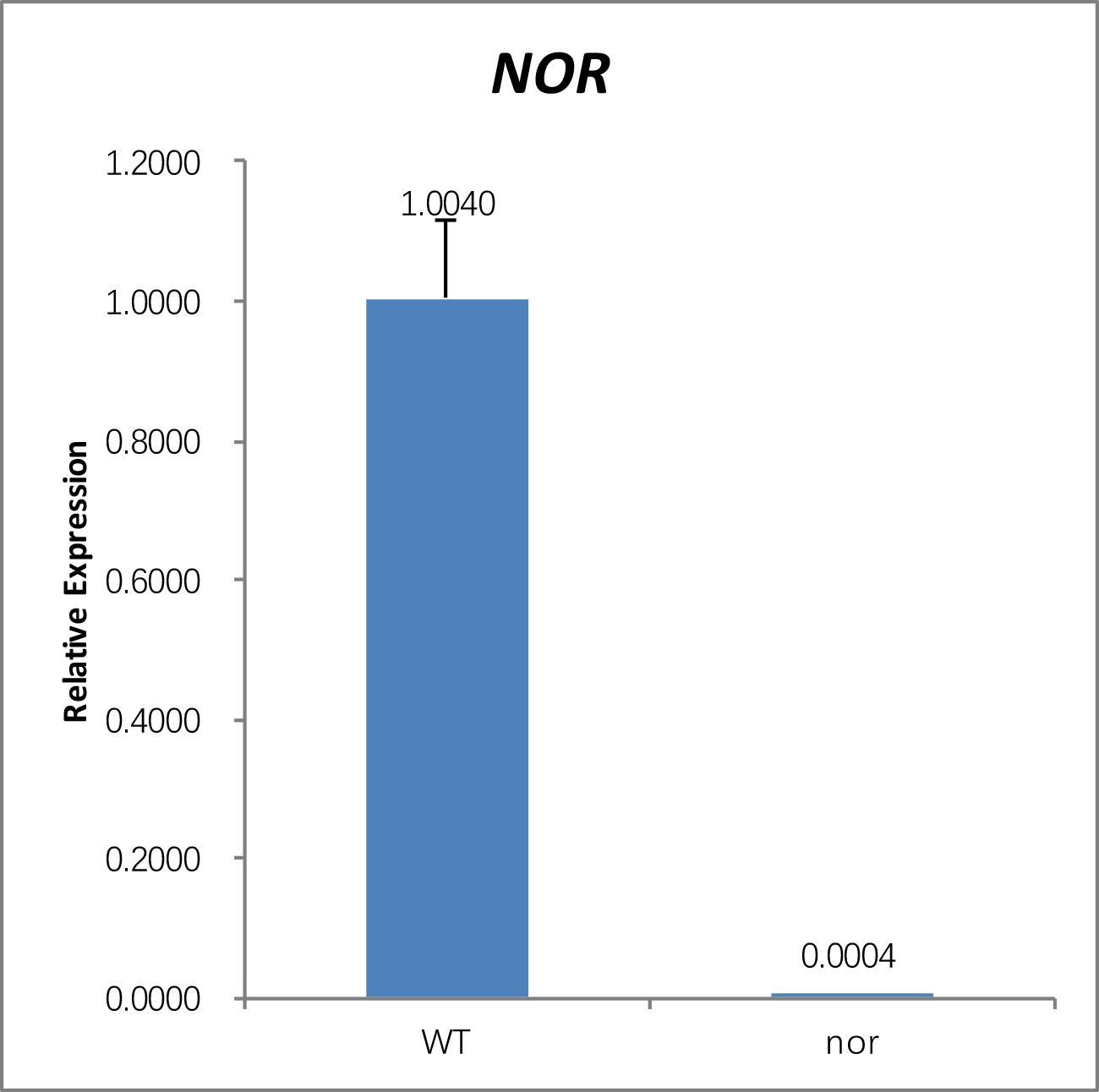

Supplement: S1 Fig — (TIF) [file pone.0164335.s001.tif]
